# Effects of E-Cigarette Refill Liquid Flavorings with and without Nicotine on Human Retinal Pigment Epithelial Cells: A Preliminary Study

**DOI:** 10.3390/ijerph182111655

**Published:** 2021-11-06

**Authors:** Shilpi Goenka, Sanford R. Simon

**Affiliations:** 1 Department of Biomedical Engineering, Stony Brook University, Stony Brook, NY 11794-5281, USA; sanford.simon@stonybrook.edu; 2 Department of Biochemistry and Cellular Biology, Stony Brook University, Stony Brook, NY 11794-5215, USA; 3 Department of Pathology, Stony Brook University, Stony Brook, NY 11794, USA

**Keywords:** retinal pigment epithelial cells, e-cigarettes, e-liquid, flavorings, nicotine, cytotoxicity

## Abstract

Smoking is an etiologic factor for age-related macular degeneration (AMD). Although cigarette smoke has been extensively researched for retinal pigment epithelial (RPE) cell degeneration, the potential for adverse effects on the retinal epithelium following exposure to flavored e-cigarette refill liquid has never been explored. In this preliminary study, we have examined the effects of 20 e-liquids (10 different flavored nicotine-free and 10 nicotine-rich e-liquids) used in e-cigarettes on the metabolic activity, membrane integrity, and mitochondrial membrane potential of RPE cells. Our results showed that of the flavors studied over the concentration range: 0.5, 1, and 2% *v/v* for a duration of 48 h, cinnamon was the most toxic and menthol was the second most toxic, while other flavors showed lesser or no cytotoxicity. The presence of nicotine augmented cytotoxicity for cinnamon, menthol, strawberry, vanilla, and banana while for other flavors there was no synergism. Together, our results demonstrate that exposure of RPE to flavored e-cigarette refill liquids caused significant cytotoxicity and may be a risk factor for the development of retinal pathogenesis, although further in-depth studies are necessary.

## 1. Introduction

Electronic cigarettes (ECs) or e-cigarettes are battery-powered nicotine delivery devices that consist of a heating coil which produces an aerosolized vapor from a liquid used in its chamber. This liquid, known as e-liquid, typically contains nicotine, flavoring chemicals, and humectants propylene glycol (PG) and vegetable glycerin (VG). ECs are believed to be less addictive and less harmful to the younger generation as compared to regular cigarettes [1,2,3,4]. The wide range of available flavors plays a central role in the popularity of ECs and many EC refill liquids have been shown to contain flavor chemicals at a total concentration that exceeded the nicotine concentration [5]. While tobacco is a traditional flavor, the youth population is more drawn towards the non-traditional flavors such as sweet, fruit, candy, or menthol, due in part, to their pleasant taste and sensory appeal as well as their capacity to mask the harshness of nicotine [6,7,8,9]. Adolescents have been shown to prefer sweet and fruity flavors while adults were shown to have a preference for menthol, tobacco and spicy flavored e-liquids [10]. To date, the market for ECs has been expanding due to its attraction to the young population and adolescents, who may select from a plethora of flavors; >7500 flavors and 466 EC brands have been identified [11]. Interestingly, the use of flavors as a marketing trend was also introduced in cigarettes in the form of flavor-containing capsule cigarettes, which have also gained popularity among the youth [12]. ECs have been shown to cause adverse effects and cytotoxicity both in vitro and in vivo studies, raising concerns about their public health and safety [13]. Moreover, the use of ECs by young adults might serve as a gateway product, leading to greater likelihood of taking up cigarette smoking in the future [14]. Multiple studies have demonstrated that the flavors in EC contribute to cytotoxicity independent of the presence of nicotine [15,16].

Retinal pigment epithelium (RPE) cells are a monolayer of cuboidal cells that constitute the outer layer of the blood–retinal barrier in the eye and are connected to Bruch’s membrane (the internal layer of the choroid) on the basal side and outer segment of photoreceptor cells on the apical side. RPE cells participate in regulating the maintenance of functions of photoreceptors as well as the phagocytosis of outer segments [17,18]. The degeneration and loss of functions of RPE underlie the primary pathological changes in age-related macular degeneration (AMD) that is the leading cause of blindness in 8.7% cases in the world [19,20]. Wet AMD is characterized by the presence of choroidal neovascularization (CNV) while in dry AMD, CNV is absent [21]. Mitochondrial damage is one of the hallmarks in the pathogenesis of AMD and mitochondria of RPE cells from AMD donors have been shown to exhibit reductions in cell functions [22,23].

Cigarette smoking has been established as a key risk factor in the pathogenesis of AMD [24,25,26]; however, there are only two reports which have explored the effects of EC on retinal tissues. In one study, the authors demonstrated that EC use in healthy men led to decreased vascular density with development of hypoxia in retinal tissue [27]. In another study, whole-body exposure of mice to vapors from unflavored nicotine-free and unflavored nicotine-rich EC showed damage to retinal microvasculature; the presence of nicotine led to further enhanced secretion of inflammatory mediators and angiogenic factors exacerbating the damage [28]. To the best of our knowledge, there is no report on the effects of EC liquid flavorings on retinal exposure to date. Hence, in this preliminary study, we have examined the impact of a limited panel of flavored e-liquids on the retinal epithelium, the cellular monolayer that provides the first line of defense against ocular toxicants. To the best of our knowledge, this is the first in vitro study to describe the effects of flavored ECs on RPE cells.

## 2. Materials and Methods

### 2.1. Materials

A total of 20 e-cigarette refill liquids (10 flavors with 18 mg/mL nicotine and 10 without nicotine) all in a PG/VG base at an 80/20 *v/v* ratio were purchased from a single online vendor (My Freedom Smokes, NC, USA; https://www.myfreedomsmokes.com, 15 September 2021). The different flavors were categorized into fruit, sweet, menthol, tobacco and other similar to a previous report [29]. The description and composition of the e-liquids are listed under Table 1. PG and VG (USP grade) were also purchased from the same vendor and mixed in an 80/20 *v/v* ratio in our laboratory and were used as a vehicle control for all experiments, while the unflavored nicotine base (in a 75/25 PG/VG vehicle) was also purchased as another control. Alamar Blue dye and Lactate Dehydrogenase (LDH) kit were procured from Thermo Fisher Scientific (Waltham, MA, USA). The mitochondrial membrane potential (MMP) assay kit was procured from Cell Signaling Technology, Inc. (Danvers, MA, USA).

### 2.2. Cell Culture

Human retinal pigment epithelial cells, ARPE-19 (CRL-2302™), were purchased from ATCC (Manassas, VA, USA) and maintained in Dulbecco’s modified Eagle medium (DMEM): F12 medium (50/50 mix) supplemented with 10% heat-inactivated fetal bovine serum (R&D Systems, Minneapolis, MN, USA) and 1% penicillin and streptomycin. The cells were grown in a 95% air, 5% CO_2_ incubator in a humidified atmosphere at 37 °C.

### 2.3. Alamar Blue Cytotoxicity Assay

Alamar Blue is a resazurin based blue-colored dye, which is non-fluorescent but upon reaction with active cellular dehydrogenases is converted to highly fluorescent pink-colored resorufin; the fluorescence can be quantitated with high sensitivity. ARPE-19 (1 × 10^4^ cells/well) were seeded in 96-well black plates (Corning Costar^®^) for 24 h followed by the addition of e-liquids at various concentrations (0.5, 1, and 2% *v*/*v*) and the cultures maintained for a 48 h duration. At the end of exposures, the culture medium was aspirated and 100 µL of fresh medium containing 10 µL Alamar Blue dye was added and the plate was incubated at 37 °C for 2 h. The fluorescence was measured using a microplate reader (Gemini EM Spectramax^®^, Molecular Devices, San Jose, CA, USA) at an excitation and emission wavelength of 570 nm and 585 nm, respectively; the relative fluorescence units (RFU) were expressed as percentage of untreated control.

### 2.4. LDH Membrane Integrity Assay

The effects of e-liquids on membrane integrity of ARPE-19 cells were monitored by the levels of LDH released from the cytosol into the culture medium. Cells (1 × 10^4^ cells/well) were pre-cultured in a 96-well plate for 24 h before the addition of e-liquids at various concentrations and the cultures maintained for a 48 h duration. After the exposure period, 20 µL of lysis solution supplied in the kit was added to one set of wells and incubated at 37 °C for 45 min; this group was used as the positive control with maximum LDH release. After this step, 50 µL of culture supernatants were combined with 50 µL of substrate mix in a new 96-well plate and incubated at room temperature for 30 min in the dark. The absorbance was recorded at 490 and 680 nm on a microplate reader. The results were reported as % LDH leakage normalized to positive control LDH levels released from lysed cells.

### 2.5. Mitochondrial Membrane Potential

ARPE-19 cells (1 × 10^4^ cells/well) were cultured for 24 h followed by the replacement of culture medium with nontoxic concentrations of various e-liquids and the cultures were maintained for a further duration of 48 h. At the end of exposure, the mitochondrial membrane potential values were assayed based on the manufacturer’s instructions. Briefly, in one set of wells (positive control) 50 µM CCCP (carbonyl cyanide 3-chlorophenylhydrazone, a classic “uncoupler” that wipes out the mitochondrial membrane potential) was added and incubated at 37 °C for 20 min. After this, cells were washed in HBSS and 200 nM tetramethylrhodamine (TMRE) dye in culture medium was added to wells; the plate was then incubated for a period of 30 min at 37 °C. After this step, the wells were aspirated, washed, and the fluorescence was read at 550/580 nm using a fluorescence microplate reader. Results are expressed as ratio of relative fluorescence of treated to that of untreated control and expressed as percentages.

### 2.6. Statistical Analysis

One-way analysis of variance (ANOVA) with Dunnett or Tukey’s post-hoc test was run using GraphPad Prism software (version 9.0). Differences were considered statistically significant at *p* < 0.05. All data are reported as Mean ± SD.

## 3. Results

### 3.1. E-Liquid Cytotoxicity to ARPE-19 Cells by Metabolic Activity Measurement

**Figure 1:**
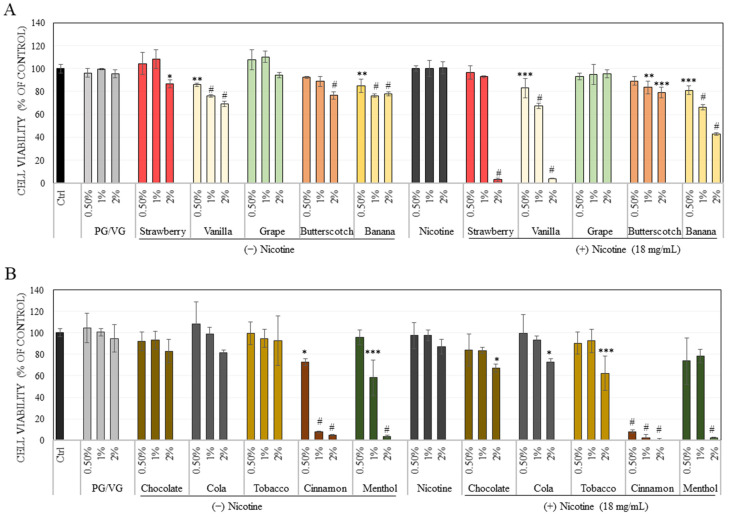
Viability of ARPE-19 cells after treatment with nicotine-free (−) and nicotine-rich (+) e-liquids of (**A**) Flavors: strawberry, vanilla, grape, butterscotch, banana, and (**B**) Flavors: chocolate, cola, tobacco, cinnamon, menthol over a duration of 48 h, as measured by Alamar Blue assay. PG/VG represents the vehicle (80/20 *v*/*v*), and Nicotine refers to unflavored base-liquid containing 18 mg/mL nicotine in 75/25 PG/VG., Data are mean ± SD of a representative experiment in triplicates out of three independent experiments, One-way ANOVA with Dunnett’s test. * *p* < 0.05; ** *p* < 0.01; *** *p* < 0.001 and # *p* < 0.0001 vs. Ctrl.

Our results showed that both the unflavored vehicle base (80/20 PG/VG) as well as unflavored nicotine base e-liquids were nontoxic to ARPE-19 cells over the concentration range (0.5–2%), while the flavored e-liquids showed cytotoxicity (Figure 1A). Our results of the effects of the five flavors: strawberry, vanilla, banana, butterscotch, and grape, on ARPE-19 cell survival are summarized in Figure 1A. The strawberry flavor induced significant cytotoxicity only at the highest concentration of 2% where it reduced viability by 13.26%, while lower concentrations were nontoxic to ARPE-19 cells. Interestingly, in the presence of nicotine, strawberry showed synergistic toxicity at 2% with a highly potent diminution in viability by 97.10%, indicative of almost no cell survival. The vanilla flavor showed a significant diminution in viability by 14.14%, 23.69%, and 30.58% at concentrations of 0.5%, 1%, and 2%, respectively. The nicotine-rich vanilla flavor suppressed viability by 16.98%, 32.57%, and 95.99% at 0.5%, 1%, and 2%, respectively. The pattern of synergistic cytotoxicity by the vanilla flavor at 2% mirrored that of the result obtained with strawberry flavor. The banana flavor significantly diminished cell survival by 15%, 23.64% and 22.22% at concentrations of 0.5%, 1% and 2%, respectively, while in the presence of nicotine, it significantly diminished cell survival by 18.93%, 33.81%, and 57.05% at 0.5%, 1% and 2%, respectively. Clearly, the banana flavor could also induce synergistic toxicity in the presence of nicotine at 2%, albeit to a lesser extent as compared to strawberry and vanilla flavors. The butterscotch flavor showed significant toxicity only at 2% where it reduced viability by 23.54% while in the presence of nicotine, the viability was significantly suppressed by 16.41% and 20.81% at concentrations of 1 and 2%. The grape flavor showed no toxicity under nicotine free or nicotine-containing conditions over the concentration range of 0.5–2%.

Our results of the effects of the remaining five flavors: chocolate, cola, tobacco, cinnamon, and menthol, on ARPE-19 cell survival are summarized in Figure 1B. The cinnamon-flavored e-liquid induced high cytotoxicity to cells at all concentrations with a significant diminution by 27.05%, 92.31% and 95.24% at concentrations of 0.5, 1 and 2%, respectively. In the presence of nicotine, cinnamon further enhanced cytotoxicity with reduction in viability by 91.88%, 97.94% and 99.79% at 0.5, 1, and 2%, respectively. Exposure to menthol-flavored e-liquid significantly lowered cell viability by 41.73% and 96.46% at concentrations of 1% and 2%, respectively. Interestingly, the presence of nicotine rescued cytotoxicity at 1% with no significant difference from untreated control, although the viability at 2% was still significantly diminished by 97.87%. The chocolate, cola, and tobacco-flavored e-liquids showed no significant change in cell viability at any concentration as compared to the control group. However, in the presence of nicotine, chocolate, cola and tobacco, lowered cell viability significantly by 32.8%, 26.99% and 37.56% at the highest concentration of 2%.

Altogether, the apparent order of cytotoxicity of nicotine-free e-liquids was cinnamon >> menthol > vanilla > banana > butterscotch > strawberry > cola = chocolate = tobacco = grape. The order of cytotoxicity in the case of nicotine-containing flavored e-liquids followed the order: cinnamon >> vanilla > menthol > banana > strawberry > tobacco > butterscotch = cola = chocolate > grape.

### 3.2. E-Liquid Effects on Membrane Integrity of ARPE-19 Cells

**Figure 2:**
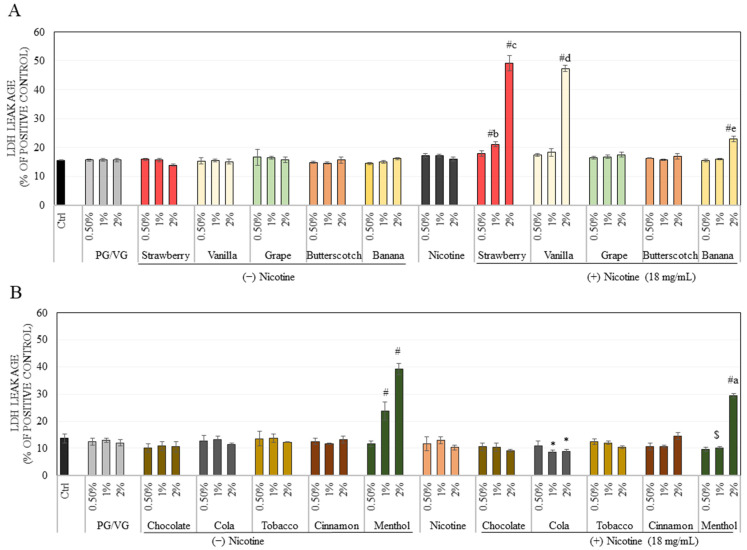
Membrane integrity of ARPE-19 cells after treatment with nicotine-free (−) and nicotine-rich (+) e-liquids of (**A**) Flavors: strawberry, vanilla, grape, butterscotch, banana, and (**B**) Flavors: chocolate, cola, tobacco, cinnamon, menthol for a duration of 48 h, as measured by LDH assay. Data are mean ± SD (*n* = 3 per group), One-way ANOVA with Tukey’s test. [* *p* < 0.05 and # *p* < 0.0001 vs. Ctrl; b: *p* < 0.0001 vs. (−) strawberry at 1%; c: *p* < 0.0001 vs. (−) strawberry at 2%; d: *p* < 0.0001 vs. (−) vanilla at 2%; e: *p* < 0.0001 vs. (−) banana at 2%; $ *p* < 0.0001 vs. (−) menthol at 1%; a: *p* < 0.0001 vs. (−) menthol at 2%].

The vehicle (PG/VG) as well as unflavored nicotine control showed no alterations in LDH levels (Figure 2A). The strawberry flavor did not affect cellular membrane integrity of ARPE-19 cells at any concentration, although in the presence of nicotine, it induced a significant increase in LDH leakage by 1.36-fold and 3.17-fold at concentrations of 1 and 2%, respectively, that was significantly higher than nicotine-free concentrations (Figure 2A).

The vanilla flavor showed a significant increase in LDH leakage only under nicotine-rich conditions at the highest concentration of 2%, where the levels were increased by 3.05-fold as compared to untreated control. The banana flavor provided a result similar to that of the vanilla flavor, except the LDH leakage was increased by a lesser amount (1.48-fold). The grape and butterscotch flavors showed no change in cell membrane integrity at any concentration either under nicotine-free or nicotine-rich conditions (Figure 2A). The menthol flavored e-liquid caused a significantly higher LDH release, while the other four flavors: chocolate, cola, tobacco, and cinnamon did not show any difference as compared to control (Figure 2B). Moreover, our results of LDH leakage for the nicotine-containing menthol flavor showed significant reduction in released LDH levels as compared to nicotine-free flavor, indicating that nicotine rescued the membrane damage incurred by menthol. Together, these results show that in absence of nicotine, menthol was the most damaging of all 10 flavors as it severely damaged retinal cell membrane. In the presence of nicotine, however, the membrane damage seemed to be partially rescued. The strawberry flavor was the most cytotoxic to cell membrane under nicotine-rich conditions followed by the vanilla and banana flavor with all the other remaining flavors exhibiting no appreciable effect.

### 3.3. Effects of E-Liquids on Mitochondrial Membrane Potential

**Figure 3:**
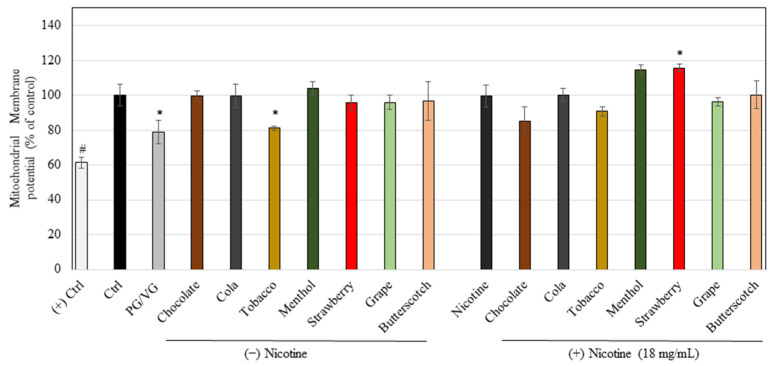
Measurement of mitochondria membrane potential in ARPE-19 cells treated with different e-liquids at a nontoxic concentration of 0.5% for a duration of 48 h. (+) Ctrl refers to the positive control in which the membrane potential has been wiped out in the presence of CCCP. Data are mean ± SD, (*n* = 3 per group), One-way ANOVA with Tukey’s post hoc test. * *p* < 0.05 and # *p* < 0.0001 vs. Ctrl.

We next compared all the e-liquids at the nontoxic concentration of 0.5%, (except cinnamon, vanilla, and banana that were excluded as they were shown to induce significant cytotoxicity to ARPE-19 cells in our previous assays) on the levels of mitochondrial membrane potential in the cells. Our results showed that the vehicle (PG/VG) at 0.5% showed a significant reduction in membrane potential levels by 21.17% as compared to the control group; the tobacco flavor continued to retain this reduction in membrane potential levels (levels reduced by 18.98%), while the other six e-liquids showed no changes in membrane potential levels as compared to the control (Figure 3). The unflavored nicotine group did not affect membrane potential levels and the presence of flavors in nicotine-rich liquids showed no significant effect, except for strawberry which, surprisingly, increased the membrane potential levels by 15.59%.

## 4. Discussion

Our results demonstrate that exposure to different flavored e-liquids induced cytotoxicity to RPE cells; the presence of nicotine augmented cytotoxicity, at least for some flavors, but only at the highest concentrations of 2%. Our results of high cytotoxicity of strawberry flavor are consistent with a previous report [30] and those of higher cytotoxicity by cinnamon and menthol are in agreement with previous reports where authors tested the effects of these flavors on embryonic and adult cells [15,31]. Similar to results of this study, cinnamon-flavored e-liquids have been shown to exhibit a consistent pattern of higher cytotoxicity than other flavors [32]. The presence of the chemical cinnamaldehyde that is known to induce cytotoxicity might account for the cytotoxicity of cinnamon flavored e-liquid [33]. Our results of LDH cytotoxicity assay of menthol showed a remarkably high release of LDH, indicative of severe membrane damage; a previous report has shown that menthol can disrupt the integrity of cellular membranes, which might be linked to the loss of tight junctions of epithelial cells [34]. On the other hand, cinnamon flavor failed to show any effects in the LDH assay, although it produced high cytotoxicity in Alamar Blue assay. One possibility is that certain chemicals or impurities in the cinnamon flavored e-liquid might have inactivated the LDH enzyme, as chemicals interfering in LDH assay has been previously documented [35]. Another possibility is that cinnamon flavor might not have induced sufficient damage to membranes to cause leakage of LDH into the culture medium, especially since the LDH assay we used only measured LDH released into the medium and not the intracellular LDH. A similar discrepancy in the cytotoxicity of e-cigarette JUUL pods were found previously, where the authors noted loss of viability but no release of LDH [36]. Our LDH cytotoxicity results in e-liquids reflect necrotic cell death but it can also reflect late apoptosis [37]. We have not elucidated the mechanisms of cell death as it was beyond the scope of this study; further studies to differentiate the modes of cell death after e-cigarette exposure are warranted. A previous study reported that exposure to nicotine-free or nicotine-containing e-cigarette vapor caused DNA strand breaks independently of nicotine in other epithelial cell lines, but the authors used longer exposures of 1 week or 8 weeks [1]. We do not expect any significant induction of DNA strand breakage in the experiments we carried out for an acute exposure of 48 h, although further studies will be necessary to confirm this.

The presence of subunits of nicotinic acetylcholine receptors in ARPE-19 cells have been reported before [38]; nicotine can bind to these receptors and modulate cellular responses [39]. Our results of absence of cytotoxicity by nicotine up to a concentration of 2% (equivalent to 2.2 mM nicotine) are in agreement with a previous study documenting that nicotine does not cause any cytotoxicity to ARPE-19 cells up to concentrations as high as 10 mM [40]; although, it should be noted that despite this, nicotine is not harmless and can impair phagocytosis of these cells at low micromolar concentrations [38]. A previous report has demonstrated that cytotoxicity of different flavored e-liquids was attributed to the concentration and total number of flavoring chemicals and not nicotine [15]. Previous reports have demonstrated that the flavor chemical aldehydes can interact with nicotine in e-liquids to form chemical adducts; reaction of these aldehydes with the PG vehicle can result in the formation of acetals [41,42]. We speculate that the synergistic toxicity in the presence of nicotine induced by exposure of cells to some flavors, which contain aldehydes could be attributed to such adducts, since both nicotine and the vehicle (PG/VG) showed no toxicity at tested concentrations. We have selected nicotine at a concentration of 18 mg/mL in this study as it is representative of moderate levels of smoking and has been used in previous studies, which compared e-liquids with and without nicotine [43,44]. Moreover, this concentration of nicotine is typically used by e-cigarette users who vape with smaller tanks [45,46]. A study showed that e-cigarettes that contain 20 mg/mL nicotine are similar to conventional cigarettes as they can achieve similar nicotine delivery rates with 1 mg delivered every 5 min [47]. It should be noted that although the typical concentration of nicotine in EC ranges from 0–36 mg/mL, JUUL pods, the newer e-cigarette devices, contain much higher levels of nicotine: 56.2 mg/mL [48] or even 75.6 mg/mL [49]. These levels are even higher than those from a pack of a conventional cigarettes, which may yield 40 mg/pack. Our results of higher cytotoxicity by some flavors (vanilla, cinnamon, strawberry, banana, and menthol) in the presence of 18 mg/mL nicotine indicate potential for retinal damage; the use of much higher nicotine levels, such as those from JUUL pods, may further exacerbate this damage, although this hypothesis warrants further exploration.

Accumulating evidence implicates mitochondrial damage in RPE as one of the factors for pathogenesis of AMD. RPE cells from older donors showed reduced mitochondrial membrane potential as compared to RPE from younger donors [50]. Our results of declines in mitochondrial membrane potential induced by the vehicle (PG/VG) alone indicate that the vehicle in EC can in itself impair functions. A previous report where mice were exposed to EC vapors showed damage to retinal microvasculature by the PG vehicle [28] while another study postulated that the vapors generated from PG produced formaldehyde on heating which led to ischemia and cellular damage in retinal vessels [27]. Although we did not observe any significant effect of flavors per se on mitochondrial membrane potential, the possibility that these flavors might contribute to oxidative stress by inducing the production of reactive oxygen species (ROS) cannot be ruled out, since it has been reported previously that flavors in EC induced the generation of hydroxyl radicals and ROS [51,52]. Further studies to explore this are warranted. We have only evaluated one endpoint of mitochondrial membrane potential in our study as it is a commonly used toxicological endpoint evaluated in other studies of toxicological exposures, hence whether flavored e-liquids might have further compromised mitochondrial biogenesis, respiration or DNA copy number were not assessed. Of note, cinnamaldehyde in flavored e-liquids has been shown to disrupt mitochondrial bioenergetics and ATP respiration in lung epithelial cells [53] and menthol flavored e-cigarette pods reduced basal and maximum respiration in mitochondria while tobacco pods had no effect on mitochondrial bioenergetics [54]. Further studies are warranted to expand upon these endpoints in RPE cells to provide a full overview of alterations in mitochondrial functions by flavored e-cigarettes for these cells.

To the best of our knowledge, there is only one recent in vivo study where the authors evaluated the effects of chronic e-cigarette vapor on mouse RPE in addition to the choroid and retina, although they did not test any flavor. Short-term e-cigarette exposures can provide initial evidence of biochemical changes in cells and can help narrow down the most important flavored e-liquids; long-term in vivo studies can then be conducted in the future to test these selected flavored e-liquids in models of chronic smoke exposure, similar to the study in the mouse model [28]. We did not evaluate the effects of the panel of e-cigarette liquids on RPE cytokine/chemokine in this study since that was not the primary focus of the study. In addition, Wang et al. [28] showed that exposure to nicotine-free or nicotine-containing unflavored e-cigarette vapors over a short-term duration of 2 weeks did not cause any significant alterations on proinflammatory cytokine levels (although the anti-angiogenic pathway was activated) in mouse retinal epithelium. It was only after a long-term exposure of 3 months, that the authors reported significant upregulation in the protein levels of cytokines VEGF, IL-1β, and iNOS. Evidently, a chronic exposure of e-cigarette is needed for effects on proinflammatory mediators. Nevertheless, future studies to address the effects of different flavored e-cigarettes on proinflammatory cytokines would be interesting. Complement activation in RPE cells can stimulate them to secrete proinflammatory cytokines (IL-6, IL-8, and MCP-1) that can contribute to AMD [55,56]. Prior studies have shown that cigarette smoke extracts can trigger the complement system in ARPE-19 cells by inducing the release of complement C3, generation of anaphylatoxin C3a, followed by the activation of alternative pathway of complement activation. The authors further showed that this proinflammatory milieu involved Nrf2 signaling [57]. Of note, the complement activation did not depend on whether the ARPE-19 cells were polarized or unpolarized in the previous study. We have used heat-inactivated serum for culture of ARPE-19 cells, hence the cell model in our experiments does not have any activated complement factors. Whether e-cigarettes can activate complement in RPE cells and predispose the cells to inflammatory milieu was beyond the scope of this study and is worthy of future investigation.

Although cinnamon and menthol flavored liquids were the most cytotoxic to RPE cells, while chocolate, butterscotch and grape showed relatively less or no cytotoxicity; the latter flavors cannot be deemed completely safe as the possibility that they might impact the functions of RPE cells, such as cell migration or phagocytosis, would need to be determined in follow-up studies. It should be emphasized that e-liquid is not the same as e-aerosol as upon heating, e-liquids are known to undergo thermal degradation and generate pyrolysis products, such as carbonyl compounds and aldehydes [58], including nanoparticles [59] which can lead to higher toxicity. Nevertheless, our pilot study highlights the significance of further investigating retinal health-related effects of flavored e-cigarettes. Future studies to test EC vapor condensates and comparison with cigarette smoke extract will also be helpful to ascertain if ECs are more harmful. Currently, only one in vivo study [28] exists where the authors demonstrated that whole body exposure of mice to cigarette or unflavored e-cigarette over 3 months caused similar adverse outcomes of upregulation in inflammatory markers in the retinal tissue. Moreover, other studies that have compared cigarette smoke and e-cigarette exposure have shown that toxicity induced by e-cigarette aerosols in bronchial epithelial cells (obtained from patients with COPD) was comparable to that of cigarette smoke [60]. Another study showed that similar to tobacco smoke, e-cigarette aerosols caused similar adverse outcomes of myocardial oxidative stress and inflammation with cardiac fibrosis in rats [61].

Multiple studies have now validated that e-cigarettes can induce side-effects on all human organs, including pulmonary, cardiovascular, and cerebrovascular systems [62,63,64]. While our current study only focused on the effects of e-cigarette liquids on RPE cells, e-cigarette exposure by dermal route or inhalation or secondhand/thirdhand exposure will also cause adverse effects in other tissues dependent on the genetics and the history of smoker; side-effects are anticipated to be higher for users who already have systemic inflammation due to other comorbidities or due to a prior history of smoking itself. Accumulating evidence validates that AMD is a local manifestation of retinal pathology due to systemic inflammation [65]. Furthermore, a report documented a two-hit model for toxicity in AMD [66], where it was discussed that the progression of AMD is dependent on the host response to exposure of toxicants; systemic oxidative stress and inflammation in other tissues will predispose the individual to develop AMD.

The use of primary RPE cells can be challenging as these cells suffer from genetic variability between donors [67] as well as limited lifespan and propensity to dedifferentiate, thereby causing loss of RPE characteristics [68]. Furthermore, the availability of primary RPE cells is limited due to shortage of human donor eyes. hTERT-RPE1, a telomerase-immortalized RPE cell line, has also been used as a model for the study of oxidative stress responses [69,70], although it was shown to differ from ARPE-19 cells in the expression of cell death and proliferation proteins [71] as well as differences in the expression of complement proteins [72]. Some of these differences might be, at least partially, related to the origin of these cells; hTERT-RPE1 is derived from the eyes of a 1-year old child while ARPE-19 is derived from the eyes of a 19-year old male. In addition, as the baseline mitochondrial function and response to oxidative stress has been shown to differ between primary and immortalized airway epithelial cells [73], it is possible that primary RPE cells and immortalized hTERT-RPE1 cells might show differential response to e-cigarettes as compared to ARPE-19 cells due to differential effects on mitochondrial function; a comparison to confirm this hypothesis would be interesting for future studies. Although ARPE-19 can be differentiated and fully polarized with melanization after 4 months of culture [74,75] using a specialized medium with 1% serum or in a shorter span of 1 week by supplementation of exogenous chemical, nicotinamide, to induce differentiation [76], the use of undifferentiated ARPE-19 cells is a relatively less time-consuming and convenient model and has been validated in several previous studies that evaluated components of cigarette exposure [38,77]. In addition, ARPE-19 cell line is a well-established model for human RPE cells [78,79] and expresses RPE cell-specific markers RPE65, cellular retinaldehyde binding protein-1 (CRALBP), and keratin-18 [80]. We acknowledge the limitation of using undifferentiated ARPE-19 cells for our assays, but as compared to well differentiated and polarized cells, undifferentiated cells offer high sensitivity and can be a good first screen model for the identification of toxicants. Moreover, the undifferentiated and unpigmented ARPE-19 cell model used by us is similar to the stage of RPE after wounding/injury during which there is partial or complete loss of pigmentation and cellular phenotype is changed to that of a regenerating epithelium. As compared to primary human RPE cells that represent young healthy adults, the ARPE-19 cell model used by us, with their lack of pigmentation and weaker tight junctions, are more representative of aged or pathologic RPE [81]. The aged RPE will be more susceptible to further injury by environmental exposure of toxicants, such as e-cigarettes. In addition, our cell model in this study more closely fits the older adults who switch to e-cigarettes from cigarette smoking. A previous study [82] showed that RPE65 expression (evaluated by RT-PCR) and CRALBP protein levels (evaluated by Western blots) were faint in 1-week ARPE-19 cultures as compared to 5-week cultures, indicating that marker expression is dependent on differentiation status of cells. We have not examined whether our results of cytotoxicity after treatments with e-cigarettes were related to a downregulated expression of aforementioned proteins in our ARPE-19 cultures, especially as we have used undifferentiated cultures. Future studies to test whether e-cigarette treatments can diminish the expression of these markers in a well-differentiated RPE are warranted.

RPE cells in vivo are known to contain melanin pigment while ARPE-19 cells lack any visible pigmentation. It would be necessary to evaluate the effects of e-liquids on pigmented RPE, especially as melanin is known to have affinity for metals [83] and it has been reported that several trace metals, such as nickel, chromium, lead, and copper are present in e-liquids [84,85,86,87]. Hence, the cytotoxicity profile of the flavors with and without nicotine will need to be determined in the physiological environment utilizing primary RPE cells for the future. Second, our results in the current study are representative of toxicity of e-liquids from a single vendor. Although, there have been reports of differences in cytotoxicity profile of the same e-liquids from different vendors or different lots from the same vendor [15,88], we have used e-liquids from a single vendor in order to minimize vendor differences. Third, we conducted our experiments using neat e-liquid as the analysis of different flavored e-liquid cytotoxicity in vitro, considered an ideal pre-screening before further testing with e-liquid aerosol can be undertaken, especially since testing aerosols is not amenable to high-throughput analysis and the variability in vapor generation poses challenges in replicability. Encouragingly, it has been demonstrated that cytotoxicity of e-liquids and their corresponding e-aerosols showed a similar response [30,88], while another report showed that in 74% of e-liquids studied, the cytotoxicity of the e-liquid could accurately predict the aerosol cytotoxicity [89]. Finally, we did not identify which flavoring chemical in the e-liquids was responsible for the cytotoxic effects observed in this study.

## 5. Conclusions

In summary, the results of this preliminary study show that e-liquids of cinnamon, menthol, strawberry, vanilla, and banana flavors are cytotoxic to RPE cells with a higher toxicity in the presence of nicotine, while e-liquids of grape, chocolate, cola, tobacco, and butterscotch flavors presented little or no cytotoxicity. Our data may help inform e-cigarette users to identify certain flavors in refill liquids that may pose risk to the retinal epithelium. Our results provide early indications that e-cigarette liquid exposure is deleterious to the retinal epithelium; however, further in-depth studies are necessary to expand our findings and evaluate if the flavorings might impact the several functions of RPE cells and might be a contributor to AMD. Future studies to evaluate the effects of different flavored e-cigarettes in primary RPE cells that are the most physiological relevant model would be necessary.

## Figures and Tables

**Table 1:** Composition of the different flavored e-liquids used in this study.

Flavor class	E-Liquid Flavor	Nicotine (mg/mL)	Vehicle (PG/VG)
Fruit	Ripe Strawberry	0	80/20
		18	
	Grape	0	
		18	
	Banana Pudding	0	
		18	
Dessert	French Vanilla	0	
		18	
	Butterscotch	0	
		18	
	Chocolate	0	
		18	
Drink	Cola	0	
		18	
Tobacco	Captain Choice Tobacco	0	
		18	
Spice	Cinnamon Roll	0	
		18	
Mint	Fresh Menthol	0	
		18	

## Data Availability

The data are available from the corresponding author upon reasonable request.
